# Tunneled Peritoneal Catheter for Refractory Ascites in Cirrhosis: A Randomized Case-Series

**DOI:** 10.3390/medicina56110565

**Published:** 2020-10-27

**Authors:** Nina Kimer, Agnete Nordheim Riedel, Lise Hobolth, Christian Mortensen, Lone Galmstrup Madsen, Mette Lehmann Andersen, Frank Vinholt Schiødt, Søren Møller, Lise Lotte Gluud

**Affiliations:** 1 Gastro Unit, Medical Division, Hvidovre University Hospital, 2650 Hvidovre, Denmark; agneteriedel@gmail.com (A.N.R.); lise.hobolth.01@regionh.dk (L.H.); christian.otto.mortensen@regionh.dk (C.M.); lise.lotte.gluud.01@regionh.dk (L.L.G.); 2 Novo Nordisk Foundation Center for Basic Metabolic Research, Bridge Translational Excellence Program, Faculty of Health and Medical Sciences, University of Copenhagen, 2200 Copenhagen N, Denmark; 3 Department of Clinical Medicine, University Hospital Køge, 4600 Køge, Denmark; logm@regionsjaelland.dk; 4 Faculty of Health and Medical Sciences, University of Copenhagen, 2200 Copenhagen N, Denmark; 5 Department of Gastroenterology, University Hospital Herlev, 2630 Herlev, Denmark; mette.lehmann.andersen@regionh.dk; 6 Abdominal Center K, University Hospital Bispebjerg, 2400 Copenhagen NV, Denmark; frank.vinholt.schiodt@regionh.dk; 7 Center of Functional and Diagnostic Imaging and Research, Department of Clinical and Nuclear Medicine, Amager-Hvidovre University Hospital, 2650 Hvidovre, Denmark; soeren.moeller@regionh.dk

**Keywords:** peritoneal catheter, PleurX, liver cirrhosis, refractory ascites, spontaneous bacterial peritonitis

## Abstract

Background and objectives: Refractory ascites markedly worsens prognosis in cirrhosis. Large volume paracentesis (LVP) is standard treatment, but complications are common. In a randomized controlled case-series, we assessed a permanent tunneled peritoneal catheter versus LVP in patients with cirrhosis and ascites. Materials and Methods: Random allocation was computer-generated, and concealment used opaque envelopes. Patients were included from January 2017 to December 2018. Inclusion criteria were cirrhosis and recurrent ascites and expected survival of more than 3 months. Results: Thirteen patients were enrolled (PleurX =6 versus LVP = 7). Seven were female, ranging in age from 51 to 80 years. No procedure-related complications occurred. Two patients died due to variceal bleeding (PleurX-group) and sepsis (LVP-group). One patient was withdrawn due to hyponatremia (PleurX-group). Two patients were withdrawn due to bacterial peritonitis and infection of unknown origin (control-group). In the PleurX-group, all patients colonized the catheter, two developed bacterial peritonitis. The most common bacterial colonization was *Staph. Epidermidis* (*n* = 4). Conclusions: In selected patients, the PleurX catheter mobilizes ascites and may be an alternative to LVP. The risk of infection should be considered in each case. The impact of colonization and risk of infections needs further investigation. The present trial does not allow for statistical conclusions.

## 1. Introduction

Ascites is one of the most common complications to cirrhosis and a frequent cause for hospitalization [1]. The formation of ascites is associated with an impaired quality-of-life and increased mortality [2]. About 5–10% of patients with cirrhosis and ascites develop refractory ascites, which is associated with a median survival of about six months [3]. Standard treatment includes repeated large volume paracentesis (LVP) and albumin infusion. LVP reduces the risk of hyponatremia and renal impairment compared to diuretics [4]. LVP is repeated when ascites re-accumulates. Complications to recurrent ascites and LVP are common and include spontaneous bacterial peritonitis (SBP), hyponatremia, and renal impairment. A subgroup of patients is eligible for a transjugular intrahepatic portosystemic shunt (TIPS). This intervention reduces the portal pressure, increases the effective arterial blood volume and has a beneficial effect on ascites and possibly survival, but also increases the risk of serious adverse events [5].

The PleurX catheter (BD Carefusion, PleurX Catheter Systems, Berkshire, UK) is a tunneled peritoneal catheter with a cuff that allows entrenchment in the subcutaneous fat [6]. The catheter allows drainage of ascites (<2 L per day) in the patients’ own home using vacuum containers [7].

In a randomized controlled study, we aimed to evaluate the beneficial and harmful effects of the PleurX catheter versus LVP and albumin in patients with non-malignant ascites due to cirrhosis.

## 2. Materials and Methods

The study was conducted as an open-label, randomized, controlled study at the Gastro Unit, medical division, University Hospital Amager-Hvidovre, Hvidovre, Denmark, with referral of patients from hospitals in the Capital Region and Region Zealand of Denmark. Participants were enrolled from January 2017 to December 2018 and followed for 6 months. The trial was approved by the Danish Health Authorities and the European Medicines Agency (Euda Med no: CIV-16-10-017324) and registered at clinicaltrials.gov (NCT03027635). The study was also approved by the Scientific Ethics Committee of the Capital Region of Denmark (H-16040179) (trial registration: clinicaltrials.gov: NCT 03027635).

All participants gave written consent to participation. The Good Clinical Practice Unit (GCP), Copenhagen University Hospital monitored the trial. The trial protocol is available as Supporting Information.

Inclusion criteria were (I) adults with cirrhosis and non-malignant recurrent ascites, defined as (a) inability to mobilize ascites despite administration of the maximum tolerable dose of oral diuretics or a daily dose of 400 mg spironolactone and re-accumulation of ascites within two weeks or (b) diuretic-related complications such as azotemia, hepatic encephalopathy, or progressive electrolyte imbalances; (II) expected survival of >3 months (by clinical assessment of the referring consultant). Exclusion criteria were (I) eligible for TIPS insertion, (II) hepatic encephalopathy or variceal bleeding within two weeks, (III) ongoing infection, (III) intraabdominal surgery within four months, (IV) an increased risk of complications as judged by the primary healthcare provider (the study protocol is available as supplementary material). All patients received Ciprofloxacin (Ciproxin) 500 mg daily. Patients were monitored for infections and all culture positive samples were repeated after 14 days for verification. In the PleurX group, ascites was drawn from the catheter and sent for culture if the patient had a contact at the hospital as a planned out-patient visit or at assessment for adverse events. An additional puncture next to the catheter was performed when colonization was suspected.

Patients were randomized with an allocation ratio of 1:1, based on a computer-generated list of random numbers. The trial was open-label due to the nature of the intervention, but allocation was concealed using serially numbered, opaque, sealed envelopes. All patients gave consent for participation, based on written and oral information, prior to inclusion.

Demographic data and standard biochemistry were collected at baseline. Insertion was performed with the patient fasting, under sterile conditions, with X-ray control and in local anesthesia by two experienced hepatologists. The placement procedure of the PleurX catheter is previously described [8,9]. All patients received periprocedural intravenous Cefuroxime 1.5 g. LVP was performed under sterile conditions, guided by ultrasound and in local anesthesia. If the volume exceeded 3 L, albumin infusion was supplied following clinical guidelines [1,2]. Patients were drained using vacuum containers of 1 L, with a maximum drainage of 2 L every other day. Vacuum bottles and sterile drainage kits containing gloves, bandages, and catheter tips were delivered directly to the patients. Home nurses managed the drainage in the patient’s own home under strict hygienic conditions. LVP was performed under sterile conditions, guided by ultrasound and in local anesthesia. In the LVP group, paracentesis was performed whenever needed throughout the trial course, with intervals between 5 and 14 days.

Health-related quality of life was assessed at baseline and monthly during the trial using the Cirrhosis-Associated ascites Symptom (CAS) score [10]. The CAS score is a validated 14-item scale addressing symptoms related to tense ascites. The score ranges from 14 to 40, and the higher the score, the worse the burden of symptoms.

The primary outcome measure was paracentesis free survival. Secondary outcome measures included cumulative number of paracenteses, cirrhosis-related complications, safety, and changes in biochemical parameters.

The primary outcome measure was paracentesis free survival. With an estimated probability of paracentesis free survival set to 0.3 at the end of the trial, an allocation ratio of 1:1, alpha 5% and power 80%, the required sample size was estimated to be a total of 28 patients (14 patients in each group). After considering the risk of participant dropout, the sample size was set to 32 patients (16 patients in each group).

We planned to include all participants randomized (intention to treat) regardless of compliance or follow-up. Patient characteristics were summarized using medians with range or proportions. We compared groups by non-parametric testing (Mann–Whitney *t*-test). When comparing results, we used last observation carried forward and performed non-parametric testing of mean differences between baseline and follow-up.

## 3. Results

**Figure 1:**
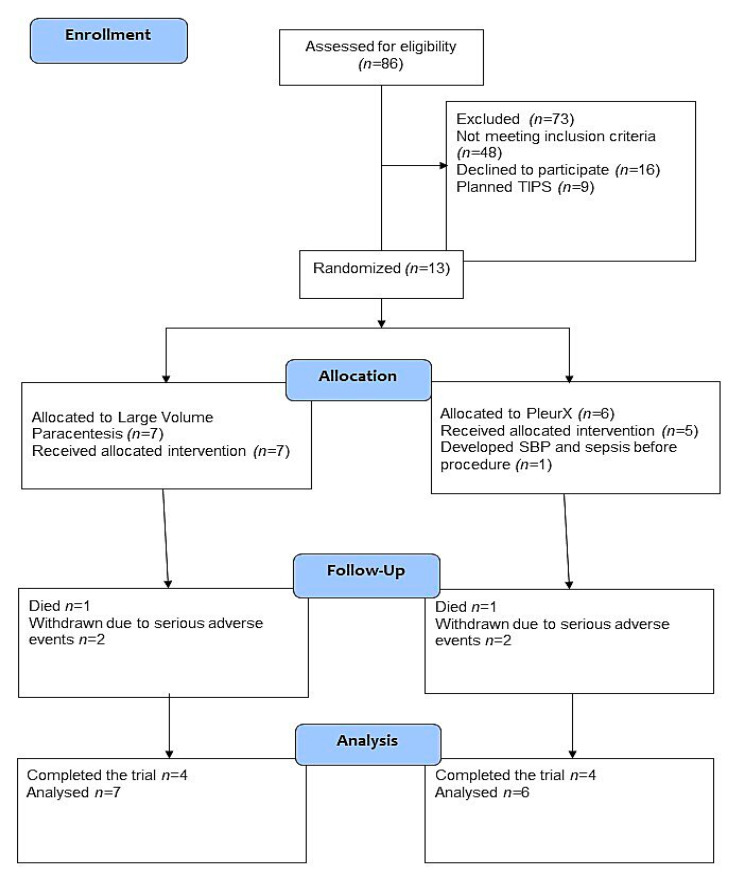
Trial flow diagram.

Eighty-three patients were screened in the trial period. Figure 1 reports patients meeting exclusion criteria and patients not meeting the inclusion criteria diuretic resistant ascites at baseline.

Recruitment rates were low due to patients not meeting the definition of diuretic resistant ascites, and to implementation of the procedure at other hospitals in the region during the study course. The screen failure ratio was high due to competing illness in a patient group with terminal liver disease, and feasibility of the study was complicated by lack of independent funding. Included patients were allocated to PleurX (*n* = 6) versus LVP (*n* = 7). The median age was 68 years (range 48–77 years); seven were male (Table 1). The median follow-up was 181 days (range 45–197 days) in the LVP group and 127 days (range 12–208 days) in the PleurX group. At baseline, two patients (both allocated to the LVP group) did not receive diuretics at inclusion due to rapid development of electrolyte disturbances when treated. The remaining 11 patients had recurrent ascites in spite of diuretic treatment in the maximum tolerated dose (Table 1). Prior to inclusion, all patients needed LVP with 6–14 days intervals.

### 3.1. Paracentesis

In the LVP group, included patients needed their first paracentesis 6 to 20 days after inclusion. During the follow up period, patients needed LVP with a median interval of thirteen days (range 8–16). The median number of LVPs ranged from 4 to 35 and the median dose of albumin administered at each LVP was 2 portions of 20 g (range 0–4 portions of 20 g).

In the PleurX group, one patient needed LVP after 56 days due to clotting of the catheter. The patient was withdrawn due to detachment of the catheter from the subcutis at 56 days. The remaining patients received drainage at home using vacuum bottles in amounts not exceeding 2 L per drainage, with a median interval of two to five days. Five patients in the PleurX group received intravenous albumin for other reasons than LVP. Albumin was given on clinical indication by house doctors due to hypotension in two patients, hyponatremia in one patient and as part of the treatment of SBP in two patients. The total median dose administered was two portions of 20 g (range 2–4 portions of 20 g).

### 3.2. Safety and Complications

One patient allocated to the LVP group died after a traumatic head injury. One patient in the PleurX group was withdrawn from the trial two days before planned insertion of the catheter due to admission with to hospital with severe sepsis and hepatorenal syndrome. One additional patient in the PleurX group was withdrawn after two months because the PleurX catheter detached from the subcutis. In the LVP group, one patient was withdrawn due to rupture of an umbilical hernia, followed by bacterial peritonitis and development of HRS, and one patient was withdrawn due to prolonged admission to hospital with HRS and HE (Table 2).

In the PleurX group, all patients developed colonization of the catheter within one to four months, but only two developed clinically verified bacterial peritonitis. The most common bacterial colonization was *Staphyloccous Epidermidis* (*n* = 4), but *Bacillus Cereus* (*n* = 2), *Enterococcus Faecalis* (*n* = 1), *Staphylococcus Hominis* (*n* = 2), and *Salmonella Dublin* (*n* = 1) were also found. In the LVP group, one participant developed bacterial peritonitis due to *Klebsiella Pneumoniae*, following the rupture of an umbilical hernia. In the PleurX group, one patient developed bacterial peritonitis with a mixed colonization (including *Enterococcus faecalis, Salmonella Dublin*, and *Acitenobacter radioresistens*). Both patients responded well to treatment with intravenous piperacillin 4 g and tazobactam 0.5 g three times daily. The PleurX drain was left in situ but recolonized after 4 weeks. We did not observe infections with multi-drug resistant bacteria.

### 3.3. Biochemistry

**Figure 2:**
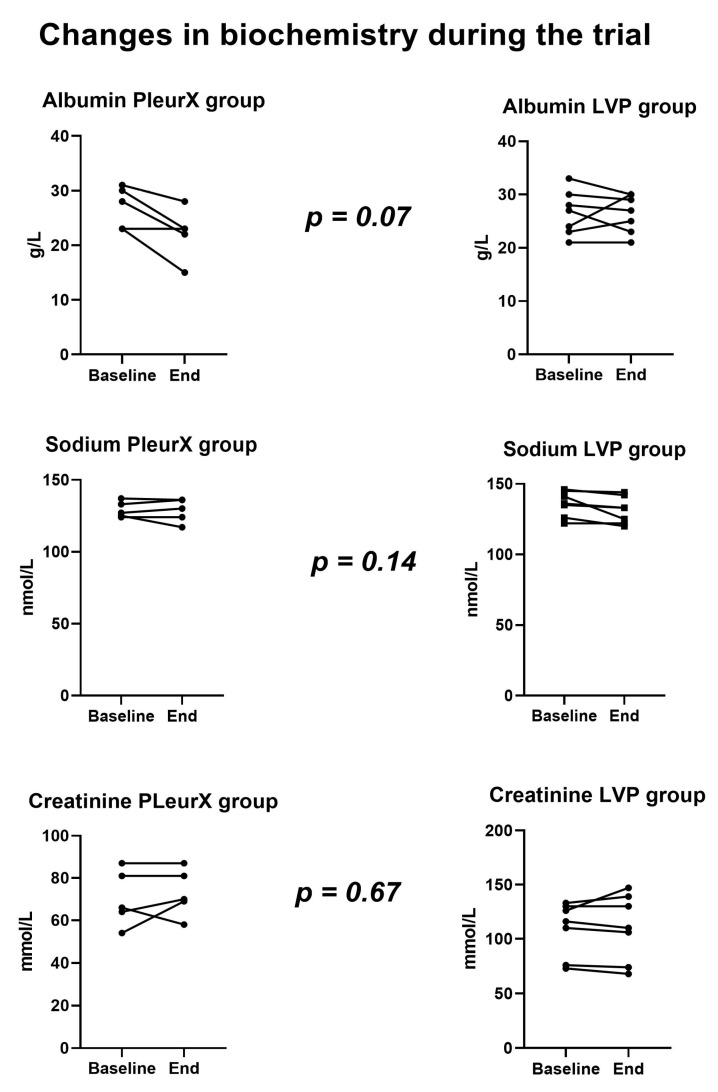
Changes in biochemistry.

We observed a moderate fall in plasma albumin levels in the PleurX group compared to the LVP group (median decrease in albumin in PleurX group was 4 g/L). One patient in the PleurX group received intravenous albumin during admission with dehydration to prevent kidney injury. Two patients received intravenous albumin as part of treatment for bacterial peritonitis. Intravenous albumin had no clear influence on the albumin levels measured during the trial, Figure 2. No significant changes in plasma sodium or creatinine levels were observed during the trial.

### 3.4. Health Related QoL (HrQoL)

The median CAS score indicated that the HrQol was relatively poor at baseline (LVP 21 points and PleurX 19 points) (Supplementary Figure S1). There was no significant difference between groups during the trial course.

## 4. Discussion

This is the first randomized controlled trial to assess the effects and safety of the PleurX catheter compared to standard treatment with LVP for patients with non-malignant ascites due to cirrhosis. Only one patient in the PleurX group needed paracentesis due to occlusion of the catheter. No procedure related complications were observed. PleurX may be effective in highly selected patients, but unfortunately the limited number of patients does not allow for statistical and robust conclusions. The trial was terminated early so clinically relevant differences may be overlooked both in terms of beneficial and harmful effects. The termination of the trial was made due to slow recruitment rates in a population of patients with terminal liver disease and many comorbidities complicating inclusion. A high screen failure ratio based on malignant ascites, variceal bleeding, HRS, or HE is evidence of a patient population with end-stage liver disease. This could mean that it will be difficult to undertake a larger randomized controlled trial evaluating efficacy of a permanent indwelling catheter in the future.

Risk of colonization and infection should be considered when using the PleurX catheter. For four patients, the PleurX catheter was a safe alternative to LVP. However, two patients were withdrawn due to serious adverse events. In one patient, hyponatremia was assessed as being associated with too frequent use of the catheter. It is therefore advised that sodium levels are checked at regular intervals.

As expected, the trial found a high risk of serious adverse events. This reflects the severity of the underlying disease [1]. Mortality in the trial corresponds to previous evidence of a poor prognosis in patients with refractory ascites [11]. Accordingly, the use of the PleurX may be considered as a palliative procedure in certain patients. If the life-expectancy is short, the benefit of the PleurX catheter may well outweigh the possible adverse events. Several retrospective studies have evaluated the safety and efficacy of the PleurX catheter [6,12,13]. The risk of bacterial peritonitis was reported in the range of eight to 42% [7,13]. Other indwelling catheters have been assessed for refractory ascites, but clinical evidence is scarce [14]. A TIPS is another alternative to LVP. The adverse events associated with the TIPS procedure may be severe and include hepatic encephalopathy, impaired liver function, and heart failure [15]. Patients with previous HE or heart disease are not eligible for TIPS placement. Novel devices have been investigated for removal of ascites. The alfapump is a subcutaneously implanted device that removes ascites from the intraperitoneal cavity and transfer it to the urinary bladder [16]. Common complications include infections, catheter clotting and dislocation of the pump, and high costs compromise the clinical application of the alfapump [17]. The Denver shunt drains ascites into the subclavian vein via a manual pump placed over the 11th or 12th rib [18]. Complication rates remain high [19].

Further studies are needed to make definite conclusions regarding the benefits or safety of the PleurX catheter. The impact of colonization on the risk of infections as well as the immunological response need further investigations. Detection of SBP should be based on both diagnostic punctures and ascites drawn from the catheter [13]. Immunosuppression is evident in the disease progression of cirrhosis [20]. It may be possible to select patients who are eligible for a tunneled catheter if future studies are able to identify valid biomarkers of immunosuppression and dysfunctional barrier functions in patients with cirrhosis.

A review from the National Institute for Health Care and Excellence (NICE) have shown similar complications rates in PleurX compared to LVP in malignant ascites, and treatment with the PleurX catheter was less costly [6]. No cost–benefit analyses are available regarding cirrhosis-associated ascites, further studies with longer follow-up are needed to evaluate the economic benefit of the PleurX catheter.

Evidence supporting the use of albumin substitution on a long-term follow-up is also warranted. Focus on quality of life and immunological dysfunctions in cirrhosis would improve our understanding of the clinical efficacy of permanent catheters in cirrhosis.

## 5. Conclusions

In conclusion, this trial suggests the PleurX catheter may be an effective treatment option in selected patients with non-malignant ascites due to cirrhosis, but the risk of infection should be considered in each case. Further evidence is needed to elucidate the risk of colonization with a permanent catheter, and the impact of colonization in patients with cirrhosis and suspected immunological dysfunction should be assessed in clinical trials in the future.

## Figures and Tables

**Table 1:** Patient characteristics

	PleurX Group (*n* = 6)	LVP Group (*n* = 7)	
Age	68 (57–77)	68 (48–75)	0.47
Gender (M/F)	1/6	5/2	NA
Child–Pugh score	9 (8–10)	9 (8–12)	0.56
Etiology (Alcohol/Nash/Hep B)	5/2/0	5/1/1	NA
Meld-sodium score	11 (8–25)	18 (11–33)	0.07
Diuretics, number of patients Furosemide daily dose (mg) Spironolactone daily dose (mg)	6 160 (0–160) 50 (0–300)	5 40 (0–160) 0 (0–200)	NA NA
Prior paracentesis interval (days)	8 (7–12)	7 (5–14)	0.75
Albumin (g/L) Sodium (mmol/L) Creatinine (µmol/L) Bilirubin (µmol/L) International normalized ratio	30 (23–32) 127 (124–137) 66 (54–81) 24 (16–49) 1,4 (1,1–1,8)	27 (21–31) 135 (122–146) 116 (73–133) 35 (7–249) 1,5 (1,1–2,0)	0.85 0.27 0.03 0.19 0.20

Results are stated in median and ranges. Non-parametric *T*-test (Mann–Whitney) is used to compare groups.

**Table 2:** Serious adverse events

	Pleurx group (*n* = 6)	Large Volume Paracentesis Group (*n* = 7)
Mortality	Terminal liver failure *n* = 1	Head trauma *n* = 1
Adverse events	Hyponatremia *n* = 2 Bacterial Peritonitis *n* = 1 Hepatic encephalopathy *n* = 1 Hypokaliemia *n* = 1 Sepsis *n* = 1	Bacterial Peritonitis *n* = 1 Hepatic encephalopathy *n* = 2 Hepatorenal syndrome *n* = 2 Infections of unknown origin *n* = 1 Variceal bleeding *n* = 1 Erysipelas *n* = 1

## Supplementary Materials

The following are available online at https://www.mdpi.com/1010-660X/56/11/565/s1, Supplementary Figure 1: Changes in Cirrhosis Ascites symptom score; Supplementary Material 1: Consort Checklist; Supplementary Material 2: Anonymized data file; Supplementary Material 3: Study Protocol.
