# Supplementary material for: Tunneled Peritoneal Catheter for Refractory Ascites in Cirrhosis: A Randomized Case-Series
Source: Medicina (Kaunas). 2020 Oct 27;56(11):565. doi: 10.3390/medicina56110565 (PMC7692861; doi:10.3390/medicina56110565)
Supplement: Supplementary file 1 [file medicina-56-00565-s001.zip › PleurX protokol v5.0 mar2018.docx]

**Tunnelated peritoneal catheter versus repeated large volume paracentesis for diuretic resistant ascites in patients with cirrhosis: An investigator initiated, open, parallel arm randomized controlled trial**

Date: March 6 2018

Short title: **PE**ri**T**oneal catheter versus **R**epeated paracentesis for **A**scites: (**PETRA**)

Protocol code: PETRA21042016

NCT03027635

Protocol version: 5.0

**Sponsor and initiator** Lise Lotte Gluud

**Investigators** Nina Kimer, Agnete Riedel, Lise Hobolth, Flemming Tofteng, Anette Dam Fialla.

**Scientific advisors** Flemming Bendtsen, Søren Møller, Marsha Y. Morgan

Contact Information

| **Primary investigator** | Nina Kimer, MD  Nina.kimer@regionh.dk |
| --- | --- |
| **Sponsor and initiator** | Lise Lotte Gluud, MD, DmSC  Lise.lotte.gluud.01@regionh.dk |
| **Institution** | Gastro Unit, Copenhagen University Hospital Hvidovre  Kettegaard Alle 30  DK- 2650 Hvidovre  Denmark  Phone Number +45 38623862  Fax Number +45 38623777 |

Recruiting Sites

Gastro Unit, medical division, Hvidovre University Hospital, Denmark.

Gastro Unit, medical division, Herlev University Hospital, Denmark.

Department of medicine, Gastroenterology, University Hospital Zealand, Køge, Denmark

Department of Gastroenterology and Hepatology, Odense University Hospital, Denmark.

Affiliations

Nina Kimer, Lise Hobolth, Flemming Tofteng, Srdan Novovic, Flemming Bendtsen, Lise Lotte Gluud. Gastro Unit, Medical Division, Hvidovre University Hospital, Denmark.

Søren Møller. Centre of Diagnostic Imaging and Research, Department of Clinical Physiology and Nuclear medicine, Hvidovre University Hospital.

Anette Dam Fialla, Aleksander Krag. Department of Gastroenterology and Hepatology, Odense University Hospital, Denmark.

Marsha Y Morgan. UCL Institute for Liver and Digestive Health, Division of Medicine, Royal Free Campus, University College London Medical School, University College, London NW3 2PF, United Kingdom.

Collaborating Units

*Analyses and investigations:* Centre of Diagnostic Imaging and Research, Department of Clinical Physiology and Nuclear Medicine, Hvidovre University Hospital.

*Biochemical analyses:* Department of Clinical Biochemistry, Hvidovre University Hospital Clinical research centre, Hvidovre University Hospital.

Time Schedule

Initiation of trial January 2017

Enrolment of patients January 2017

End of inclusion period January 2021

End of follow up June 2021

Assessment and data analysis April 2024

Publication of results September 2024

Protocol Summary

| **Background** | Ten percent of patients with cirrhosis develop ascites. In 90% of patients, ascites can be treated with diuretics. The management of the remaining 10% with diuretic resistant ascites is challenging. Symptoms including abdominal pain, dyspnoea, nausea, vomiting, and anorexia have a detrimental impact on the quality of life. Repeated large volume paracentesis provides only temporary improvement of symptoms. |
| --- | --- |
| **Hypothesis** | Insertion of a tunnelated peritoneal catheter (PleurX) allows repeated intermittent small volume fluid drainage at home. The treatment may improve the management of ascites and have a beneficial effect on the quality of life. |
| **Objectives** | To evaluate the beneficial and harmful effects of the peritoneal catheter (PleurX) versus repeated large volume paracentesis for patients with cirrhosis and diuretic resistant ascites. |
| **Design** | Investigator initiated, randomised, single blind, parallel arm, controlled trial. |
| **Blinding** | Due to the nature of the intervention and the primary outcome measure, we are unable to conduct the trial with blinding of the patients, the investigators or use blinded outcome assessment. |
| **Interventions** | Tunnelated peritoneal (PleurX) catheter versus large volume paracentesis. All patients will receive ciprofloxacin to prevent spontaneous bacterial peritonitis. |
| **Population** | We will include 32 adult patients with cirrhosis of any aetiology and diuretic resistant ascites. |
| **Sites** | Primary site: Gastro Unit, medical division, Hvidovre University Hospital, Denmark,  Gastrounit, medical division, Herlev University Hospital, Denmark, Department of medicine, Gastroenterology, Køge Hospital, Denmark, Department of Gastroenterology and Hepatology, Odense University Hospital, Denmark. |
| **Inclusion period** | 48 months. |
| **Follow up** | The total duration of follow up is six months. |
| **Primary Outcomes** | The primary outcome is paracentesis free survival. |
| **Secondary Outcomes** | Secondary outcomes include cumulative number of paracentesis, cirrhosis-related complications, safety, quality of life, changes in metabolic and biochemical parameters. |

|  | trial flow illustrated in flow chart | | | | | | |  |
| --- | --- | --- | --- | --- | --- | --- | --- | --- |
| **Prior to enrolment** | Identify eligible patients with ascites. Collect informed consent. | | | | | | |  |
|  |  |  |  | | |  |  |  |
| **Visit 1** | **Day 0:**  Standard biochemical analysis, *Special biochemical analysis,*  assessment of ascetic fluid | | | | | | |  |
|  |  | | |  |  | | |  |
|  | PleurX 16 patients | | |  | Repeated paracentesis 16 patients | | |  |
|  |  | | |  |  | | |  |
| **Visit 2** | **Day 1:** Collect baseline data. Quality of life, Standard biochemical analysis*,* Nutritional parameters (weight, handgrip strength, BMI, *DXA scan),* | | | | | | |  |
|  |  |  |  | | |  |  |  |
| **Visit 3** | **1 month:** Assessment of ascetic fluid*, Quality of life, Standard biochemical analysis, Nutritional parameters (weight, handgrip strength, BMI) | | | | | | |  |
|  |  |  |  | | |  |  |  |
| **Visit 4** | **2 months:** Assessment of ascetic fluid*, Quality of life, Standard biochemical analysis, Nutritional parameters (weight, handgrip strength, BMI) | | | | | | |  |
|  |  |  |  | | |  |  |  |
| **Visit 5** | **3 months:** Assessment of ascetic fluid*, Quality of life, Standard biochemical analysis, Nutritional parameters (weight, handgrip strength, BMI, *DXA scan),* | | | | | | |  |
|  |  |  |  | | |  |  |  |
| **Visit 6** | **4 months:** Assessment of ascetic fluid*, Quality of life, Standard biochemical analysis, Nutritional parameters (weight, handgrip strength, BMI) | | | | | | |  |
|  |  |  |  | | |  |  |  |
| **Visit 7** | **5 months:** Assessment of ascetic fluid*, Quality of life, Standard biochemical analysis, Nutritional parameters (weight, handgrip strength, BMI) | | | | | | |  |
|  |  |  |  | | |  |  |  |
| **Visit 8** | **6 months:** Final assessment. Assessment of ascetic fluid*, Quality of life, Standard biochemical analysis, Nutritional parameters (weight, handgrip strength, BMI, *DXA scan), n* | | | | | | |  |

*Parameters written italic are assessed only at visit 1 or 2, 5 and 8.*

*Patients randomized to regular large volume paracentesis: Ascites fluid will be analyzed at each paracentesis. Patients randomized to PleurX catheter: Ascites fluid will be analyzed at visit 1 and visit 3-8.

Background

Ascites is one of the most common complications to cirrhosis and a frequent cause for hospitalisation.^1 2^ Ten percent of patients with cirrhosis will develop ascites within ten years after the initial diagnosis.^3^ The formation of ascites is associated with an impaired quality-of-life and increased mortality.^1 4^ Fifty percent of patients with ascites die within five year of the diagnosis. Important prognostic predictors include hyponatraemia, increased serum creatinine, and a low arterial pressure.^5^ About 90% of patients with cirrhosis and ascites respond to treatment with diuretics.^6^ Progression to diuretic-resistance is generally an irreversible process and the management of the remaining 10% of patients is challenging.^2 7^ When patients develop diuretic resistant ascites, the median survival is about six months.^8 9^ Complications associated with reduced survival include *spontaneous bacterial peritonitis,* ^10-13^ h*yponatremia,* and *renal impairment*.^11 14^

Diuretic-resistant ascites is characterized by neuro-humoral activation of the sympathetic and renin-angiotensin-aldosterone systems as well as a low urinary excretion of sodium.^15-17^ Angiotensin II and norepinephrine increase sodium reabsorption in the proximal tubule and aldosterone in collecting tubules. The changes add to ascites formation and increase the risk of renal impairment and electrolyte disturbances. Increased levels of vasopressin result in an increased water retention relative to sodium retention, which means that patients often develop hypervolemic hyponatremia. The changes in the nervous system and vasopressors combined with the haemodynamic changes associated with portal hypertension increase the risk of renal impairment. The pathophysiology also involves an increased production of nitrous oxide and other vasodilators, which reduce the systemic vascular resistance.^18-20^ In the early phases, the cardiac output is increased to compensate for the changes.^21-23^ As the disease progresses the cardiovascular compensation becomes insufficient, ultimately leading to a decreased cardiac output and a reduced systemic blood pressure. The changes combined with increased activation of the renin-angiotensin system have deleterious effects on the kidneys. The treatment with diuretics further increases the risk of hyponatriemia and renal impairment, which are frequent causes of treatment discontinuation. *Hepatorenal syndrome* is a reversible renal failure that occurs in patients with cirrhosis and ascites.^2 6 20^ The diagnosis requires exclusion of other causes of renal failure such as acute tubulo-interstitial nephropathy and dehydration. The pathophysiology involves renal hypoperfusion associated with cirrhosis and portal hypertension. The decreased cardiac output and arterial vasodilation activate the systemic vasoconstriction as previously described. Infections such as spontaneous bacterial peritonitis may exacerbate the changes and therefore the risk of developing renal failure.^24^ The changes can lead to renal vasoconstriction and hepatorenal syndrome. In spite of treatment, the median survival is only three months.^25^

Malnutrition is a common complication in patients with end-stage cirrhosis and ascites.^26 27^ The frequency of malnutrition is directly associated with the severity of the underlying liver disease and extent to which malnutrition is an independent predictor of mortality is debated. Diuretic resistant ascites has a negative impact on the nutritional status and metabolism.^28^ Malnutrition has detrimental effects on protein turnover^29^ and the immune system with increased susceptibility to infections^30 31^ and increase morbidity and mortality in cirrhosis.^32 33^ In patients with cirrhosis, the nutritional status is an independent predictor of impaired quality-of-life.^34^ In patients with malnutrition and ascites, insertion of a transjugular-intrahepatic shunt (TIPS) improves the body composition and the metabolic profile.^35^

Interventions for patients with diuretic resistant ascites

*Liver transplantation* should be considered in diuretic resistant ascites. Contraindications could prevent transplantation and several countries have a shortage of donors. Conservative interventions are used for patients on the waiting list and patients who are not eligible for transplantation.

*Reducing the dietary salt intake* decreases hyponatriemia in up to 20% of ascites.^36^ The clinical effect of sodium restriction is not assessed in controlled trials, but general recommendations are that a moderate restriction of the dietary salt intake to less than 120 mmol per day should be considered. A reduction of the fluid intake may also be considered if patients have hypervolemic hyponatremia. The dietary changes should be introduced carefully while considering the risk of worsening of the malnutrition that is common among patients with end-stage cirrhosis.

Patients with ascites have increased tubular sodium reabsorption and hypervolaemia.^37 38^ Increased aldosterone levels play an important part of the pathogenesis and the aldosterone antagonist *spironolactone* is the most efficient diuretic.^2 7^ The use of the loop diuretic furosemide is debated. Randomised clinical trials including patients with moderate ascites have reached different conclusions regarding the benefit of combination. ^39 40^ There are no trials evaluating the effect of diuretics on refractory ascites, but the combined evidence from the trials suggest that combination therapy is more effective.^2^ However, patients with diuretic-resistant ascites have a considerable risk of clinically important electrolyte disturbances and renal impairment, which often lead to treatment discontinuation. Increasing evidence suggests that midodrine, which is an oral vasopressin analogue may reduce the adverse effects of diuretics.^38 41^ So far, routine use of midodrine is not recommended and the drug is unavailable in several countries including Denmark.

The *TIPS* is a stent shunt between a hepatic vein and the portal vein.^35 42-46^ The intervention reduces the portal pressure, increases the effective arterial blood volume and as a beneficial effect on ascites and possibly survival. The adverse events may be severe and include hepatic encephalopathy and impaired liver function. Patients with previous encephalopathy and patients with heart disease are not eligible for TIPS placement.

Large-volume paracentesis and albumin infusion is the treatment of choice for diuretic resistant ascites.^2 7^ Controlled trials have found that large volume paracentesis combined with albumin infusion is more effective and safer than diuretics. The evidence suggests that large volume paracentesis reduces the risk of hyponatremia, renal impairment, and hepatic encephalopathy compared to diuretics.^2 6 7^ The risk of local complications such as hemorrhage and bowel perforation is very low.^47^ The main complication is related to the removal of large volumes of ascitic fluid and is known as post-paracentesis circulatory dysfunction. The condition is associated with rapid re-accumulation of ascites, hepatorenal syndrome, dilutional hyponatremia due to water retention, increasing portal pressure, and increased mortality. Infusion of albumin is an effective intervention that prevents the development of post-paracentesis circulatory dysfunction. The effect of the combined treatment with large volume paracentesis and albumin is temporary and patients require diuretics to prevent re-accumulation of ascites. In a study including 10 patients with cirrhosis and tense ascites,^48^ large volume paracentesis had a beneficial effect on splanchnic circulation and the porto-collateral blood flow. After one hour, the cardiac output was increased and the wedged hepatic venous pressure, the hepatic venous pressure gradient, and the azygos blood flow decreased. A similar study including 12 patients,^49^ found that paracentesis resulted in both cardiovascular and humoral changes. The clinical effect of these changes is not established.

The paracentesis should be performed under sterile conditions. The evidence concerning the risk of infections following paracentesis is scarce. There are no randomised controlled trials that specifically asses the question. Based on trials comparing large volume paracentesis versus TIPS, the risk of infections is very low.^45 46^ Based on a Cochrane systematic review,^45^ two randomised controlled trials reported the number of patients who developed infections after the procedure. One trial comparing TIPS versus large volume paracentesis with albumin,^43^ found that four of 35 patients (11%) in the paracentesis group developed peritonitis. The second trial compared medical therapy defined as a combination of salt restricted diet, diuretics, and large volume paracentesis alone or combined with TIPS.^42^ In the control group, two of 48 patients (4%) developed peritonitis. The number of patients with peritonitis in the TIPS group was four, but the paper does not describe the number of patients that required paracentesis.

Based on the risk of complications associated with insertion of a TIPS and the potential complications and temporary clinical response to large volume paracentesis, a randomised trial including 40 patients with cirrhosis, evaluated effects of the *Automated Low-Flow Ascites (ALFA) pump*.^50^ The ALFA pump moves ascitic fluid from the peritoneal cavity into the urinary bladder. The trial found that the pump removed 90% of the ascites and reduced the median number of large volume paracentesis per month. Adverse events included problems related to the insertion of the bladder catheter, bleeding, and infection. At present, the pump is not recommended for routine use, partly due to the high costs associated with the intervention.

Insertion of a tunnelated peritoneal catheter has been shown to be an effective and safe treatment for malignant ascites.^51^ The catheter (PleurX) allows intermittent fluid drainage at home using vacuum containers. Potential benefits include the ability to improve the control of symptoms and reduce the need for large-volume paracentesis. A study including 38 patients with malignant ascites found that two patients (5%) developed peritonitis after insertion of the catheter. The study did not include a control group. A review from the National Institute for Health Care and Excellence (NICE) found eight observational studies reporting complications to the PleurX.^51^ Six studies were case series. The number of catheters requiring removal ranged from 0% to 7.5% and the total number of complications ranged from 0% to 59%. Reasons for removal of the device included infection, leakage of ascitic fluid, and loculated ascites. Ten of 172 patients (6%) developed peritonitis. The infections required removal of the catheter in two patients. Eight patients were treated successfully with antibiotics. Other complications included ascitic fluid leakage and occlusions. Only one study compared complications associated with the PleurX catheter and large volume paracentesis.^52^ The study used a retrospective design and the maximum duration of follow up was 41 months. One patient treated with the PleurX catheter developed peritonitis. The overall complication rate in the two comparison groups was 7.5%. There are no controlled studies evaluating the PleurX for non-malignant ascites. A retrospective study evaluated the risk of peritonitis following insertion of a PleurX catheter for diuretic resistant ascites in cirrhosis.^53^ The study included 149 patients and data from 200 catheter placements. Each placement is considered a separate event. Accordingly, some patients are included in the analysis more than once. In total, peritonitis was diagnosed after 19 placements (10%). Most were culture-negative. There are no data regarding the potential beneficial effects of the intervention or the use of antibiotics, but the authors report that they found no predictors of infection or mortality. The study does not include a control group.

Rationale

Repeated large volume paracentesis allows only temporary mobilisation of ascites and relief of symptoms. Insertion of a tunnelated peritoneal catheter (PleurX) allows repeated intermittent fluid drainage at smaller volumes. The PleurX catheter may therefore have beneficial effect on the management of ascites and improve quality-of-life as well as nutrition.

Objectives

To evaluate the beneficial and harmful effects of the PleurX catheter compared with large volume paracentesis.

Interventions

PleurX catheter compared with large volume paracentesis. Both interventions (PleurX catheter and large volume paracentesis) will be combined with albumin and the antibiotic ciproxin 500 mg once daily. The treatment with ciproxin 500 mg once daily will continue until the end of the trial. The PleurX cathether is approved for permanent placement. After completion of the trial, patients may choose to keep the PleurX or to have it removed.

*PleurX catheter*: The PleurX catheter is a tunnelated peritoneal catheter, designed for permanent placement in the peritoneal cavity (see appendix 6, pictures of the equipment). The catheter is placed by a trained, specialised physician under sterile conditions and with access to x-ray transillumination. All investigators involved in the placement of the PleurX catheter are specialised hepatologists with extensive experience in the management and observation of patients with refractory ascites including experience in the use of the PleurX and large volume paracentesis through their clinical work.

The catheter is 50 cm in length and is placed in the peritoneal cavity (Appendix). A small incision (approximately 1.5 cm) allows the catheter access to the peritoneal cavity from where fluid can be drained. The catheter is placed in a 5 to 8 cm subcutaneous tunnel and penetrates the skin at another incision. Initially, the catheter is attached to the skin with two sutures. A special cuff on the catheter allows entrenchment of the catheter in the subcutaneous fat. After 7-10 days, the incisions are healed and the catheter is simply wrapped in a soft bandage on the abdominal skin.

Once the catheter is placed, drainage of ascites can be done using vacuum bottles connected to the catheter (see Appendix F). A home nurse will be trained in the drainage procedure and patient monitoring by specialised company staff from Vingmed. (http://www.carefusion.com/our-products/interventional-specialties/drainage/about-the-pleurx-drainage-system/patient-information-pleurx-system/how-to-drain-at-home). The home nurse will ensure that only a maximum of two litres of ascites fluid is removed at a time.

*Large volume paracentesis*: Removal of more than five litres of ascites is defined as large volume paracentesis. Initially, an abdominal ultrasound is performed. This allows an estimate of the ascites volume and distance from the skin to the ascitic fluid. The procedure is performed with the patient lying on his back in a slightly recumbent position toward the site of paracentesis. The ultrasound is used to ensure adequate insertion of the needle in the ascitic fluid. Generally, the insertion site is inferior to umbilicus 2-3 fingerbreadths below the umbilicus. The area is cleaned with betadine in and sterile drapes applied. Using sterile technique, local anesthesia is applied to the area from the skin to the peritoneum. A small incision is made (about 1.5 cm). Subsequently, the catheter, which contains a needle is inserted. Once fluid is aspirated, the needle is removed from the plastic catheter. The catheter is then attached to the catheter bag. After the procedure, the patient remains in hospital for observation until the fluid is drained. Vital signs are checked and the amount of fluid drained is registered. After removal of 4 liters, 25 cc of albumin (25% solution) is given for every following 3 liters of ascitic fluid removed.

Potential Risks

Risks associated with peritoneal catheter and large volume paracentesis

The risks associated with insertion of a peritoneal catheter and large volume paracentesis include pain at insertion of the drain, ascites leakage, haemorrhage, hypotention, electrolyte depletion, protein depletion, peritonitis, and bowel perforation. Based on previous evidence,^51 53^ the combined risk of these adverse events is 10% in both intervention groups.

*Pain*: Infiltration of the puncture site with a local analgesic will be used before insertion of the peritoneal catheter and large volume paracentesis. The administration of local anaesthetic may be pain free or be associated with a sharp sudden pain.

*Haemorrhage*: Less than 10% develop a local haematoma at the puncture site. More severe bleeding including peritoneal haemorrhage is described in the literature, but is very rare occurring in less than one percent.

*Hypotention, electrolyte and protein depletion*: The main risk associated with removal of ascites is hypotention, electrolyte and protein depletion. The risks occur after removal of at least four litres of fluid. Administration of albumin reduces the risk of these complications and is administered after removal of the first four litres (as described above). The risk is associated with the amount of fluid removed. Using the peritoneal catheter allows removal of smaller amounts of ascites (< 2 litres) and the risks of these complications are therefore negligible. To avoid electrolyte and protein depletion, patients in both interventions will be monitored with regular blood tests.

*Peritonitis*: Patients with diuretic resistant ascites have an increased risk of developing bacterial peritonitis. In addition, both interventions are associated with an increased risk of peritonitis. To reduce the risk of peritonitis, we will administer ciproxin prophylaxis to all patients.

*Bowel perforation*: Both interventions are associated with a potential risk of bowel perforation. The complication is very rare affecting less than one percent.

Risks associated with assessments of outcomes

The risks associated with blood sampling include local haematoma and pain.

The body composition will be assessed using dual-energy X-ray absorptiometry (DXA scan). The radiation associated with the scan corresponds to a conventional chest X-ray (0.02 mSv).

Potential Benefits

The potential benefits of the PleurX drainage system include improved management of ascites, quality-of-life, symptom management, and improved nutrition.

Primary Outcome Measure

- Paracentesis free survival

Secondary outcome measures

- Cumulative number of paracenteses
- Quality of Life
- Adverse events (as defined below under definition of adverse events and monitoring).

The assessment of outcome measures is described in Appendix A.

STUDY DESIGN

This trial will be conducted in compliance with the protocol, EN ISO 14155 ”Clinical investigation of medical devices for human subjects – Good clinical practice” and the applicable regulatory requirements.

Patient inclusion criteria

- Cirrhosis of any aetiology;
- Diuretic resistant ascites defined as i) an inability to mobilise ascites (minimal or no weight loss) despite administration with the maximum tolerable doses of oral diuretics or a daily dose of spironolactone 400 mg and re-accumulation of fluid after therapeutic paracentesis within two weeks or ii) diuretic-related complications including (but not limited to) azotemia, hepatic encephalopathy, or progressive electrolyte imbalances;
- Able to read and understand Danish;
- Signed and dated informed consent form;
- Willing to comply with all study procedures and be available for the duration of the study;
- Male or female of any age;
- Age at least 18 years;
- Expected survival at least three months.

Patient Exclusion criteria

- Participants eligible TIPS, and planned for procedure within two months;
- Serum creatinine levels above 135 umol/L;
- Overt hepatic encephalopathy in the two weeks before randomization;
- Ascites due to other causes than cirrhosis such as: malignant disease, congestive heart failure, end-stage renal disease, pancreatitis, or Budd-Chiari (hepatic vein thrombosis), or chylous ascites;
- Ongoing intra-abdominal infection (peritonitis) or active systemic or local infections, such as urinary tract infection or pneumonia;
- Participation in a clinical study that may interfere with participation in this study;
- Evidence of extensive ascites loculation;
- Variceal bleeding within two weeks before randomisation;
- Intraabdominal surgery within four months before randomisation;
- Spontaneous bacterial peritonitis (neutrophil count>250/µl within 24 hours of randomization);
- Patients with an increased risk of procedure related complications as judged by the primary healthcare provider.

Strategies for Recruitment and Retention

Potentially eligible patients will be identified through daily assessments of patients treated at the clinical sites.

Sponsor and primary investigator will provide thorough information about the study design to specialised hepatologists at the recruiting sites. The trained physicians will then be authorized to identify eligible patients in their clinical practice. After conveying adequate study information to possible study participants the patients can either sign informed content or the physician can schedule an inclusion consultation with an investigator at primary site. If screened patients have no interest in taking part in the project we will register patient data in a screening log.

Two investigators will independently assess if patients fulfil all of the inclusion and none of the exclusion criteria. We will attempt to gather data on patients who are excluded from the study and data on patients who are too ill to participate in follow up assessments. We will collaborate with primary health care providers including at-home nurses in order to collect valid information about adverse events and other clinical outcomes.

Subject Withdrawal

Patients may withdraw voluntarily from the study at any time upon request. The investigator can also withdraw patients from the trial if the patient becomes unstable or there are safety concerns. If patients wish to withdraw or are withdrawn from the study, we will attempt to collect data on adverse events and other clinical outcomes based on hospital or health care records.

Premature Termination or Suspension of Study

This study may be suspended or prematurely terminated if there is sufficient reasonable cause. Written notification, documenting the reason for study suspension or termination, will be provided by the suspending or terminating party. Circumstances that may warrant termination include, but are not limited to: Determination of unexpected, significant, or unacceptable risk to subjects.

Randomisation procedure

The allocation sequence will be generated based on simple 1:1 randomisation using computer generated random numbers. The allocation will be concealed in serially numbered opaque sealed envelopes kept at a central independent randomisation office at the Gastro Unit, Hvidovre Hospital by a person not otherwise involved in the trial in order to ensure that the allocation of the next patient is concealed until the moment of randomisation. The office will be contacted by phone by recruiting physicians.

blinding

Due to the nature of the intervention, it is not possible to conduct the trial with blinding of patients or investigators.

TRIAL FLOW

prior to enrolment

We will screen potentially eligible patients admitted to the clinical sites based on daily generated lists of patients referred to in-hospital management or outpatient clinics. Patients with cirrhosis and diuretic resistant ascites will also be identified from records of patients followed in the outpatient clinics. Initial assessment of background information will be performed based on hospital records including demographics, primary diagnosis, co-morbidities, medical treatments, and surgical procedures. Patients who are potentially eligible will be approached by an investigator.

Patient history will then be re-evaluated to determine eligibility based on inclusion/exclusion criteria.

Potential participants will be provided with written and oral information as described below.

Visit 1 (Day 0)

- Patients have been fasting overnight.
- Ensure the informed consent
- Collection of:
- blood tests for standard biochemical analysis and special biochemical analysis (biobank)
- control of ascites (amount of ascites assessed using abdominal ultrasound)
- Conduct the allocated intervention procedure (PleurX insertion or large volume paracentesis)
- Patients allocated to PleurX are informed about the handling of the PleurX catheter including the importance of removing a maximum of two litres of ascitic fluid. The information will be repeated by the nurse at the home visit.
- Collect information about procedure related complications and adverse events.
- Ensure that the procedure is complete (ascitic fluid is mobilised using the PleurX catheter or large volume paracentesis).

Visit 2 (Day 1)

- Collection of information about:
- adverse events
- quality of life
- Collection of:
- blood tests for standard biochemical analysis
- weight and height
- hand grip strength
- Assessment of:
- Nutritional parameters (weight, handgrip strength, BMI, DXA scan)

Visit 3 (Day 30)

Outpatient visit including collection of information about control of ascites (amount of ascites assessed using abdominal ultrasound and number of paracenteses since the last visit), adverse events and quality of life.

Collection of blood tests for standard biochemical analysis, measurement of weight and hand grip strength and calculation of BMI.

Visit 4 (Day 60)

Outpatient visit including collection of information about control of ascites (amount of ascites assessed using abdominal ultrasound and number of paracenteses since the last visit), adverse events and quality of life.

Collection of blood tests for standard biochemical analysis, measurement of weight and hand grip strength and calculation of BMI.

Visit 5 (Day 90)

Patients have been fasting overnight.

Outpatient visit including collection of information about control of ascites (amount of ascites assessed using abdominal ultrasound and number of paracenteses since the last visit), adverse events and quality of life.

Collection of blood tests for standard biochemical analysis, measurement of weight and hand grip strength and calculation of BMI.

Visit 6 (Day 120)

Outpatient visit including collection of information about control of ascites (amount of ascites assessed using abdominal ultrasound and number of paracenteses since the last visit), adverse events and quality of life.

Collection of blood tests for standard biochemical analysis, measurement of weight and hand grip strength and calculation of BMI.

Visit 7 (Day 150)

Outpatient visit including collection of information about control of ascites (amount of ascites assessed using abdominal ultrasound and number of paracenteses since the last visit), adverse events and quality of life.

Collection of blood tests for standard biochemical analysis, measurement of weight and hand grip strength and calculation of BMI.

Visit 8 (Day 180; final assessment in the trial)

Patients have been fasting overnight.

Outpatient visit including collection of information about control of ascites (amount of ascites assessed using abdominal ultrasound and number of paracenteses since the last visit), adverse events and quality of life.

Collection of blood tests for standard biochemical analysis, measurement of weight and hand grip strength and calculation of BMI. The PleuX tunnelated catheter is removed if the subject decides that he/she would like the PleurX catheter removed after the final visit.

Unscheduled/Intermediate Visits

If patients are admitted between scheduled visits information about adverse events will be collected.

Medical device and labelling

The medical device tested is the PleurX tunnelated catheter (Pleurx Peritoneal Catheter System): CareFusion Pleurx Catheter Systems: K1 13854. The PleurX peritoneal catheter and sub devices relevant for the drainage procedure are listed in appendix: “Peritoneal Catheter Mini Kit Instructions for use_en”. Following precautions will be made when handling the medical device:

- The PleurX Tunnelated Peritoneal Catheter is approved for permanent placement and catheter implantation is regular clinical practice prior to initiation of this study. Consequently the registration of medical device handling will include noting device LOT number in patient’s medical record and in the corresponding CRF.
- All peritoneal catheters are labeled with following information:
  - Manufacturers name and address: CareFusion, 75 North Fairway Drive, Vernon Hills, IL 60061 USA.
  - Exact description of package content.
  - Package content is STERILE.
  - LOT number.
  - Expiring date.
  - For single use only.
  - Warnings:
    - Do not use if package is damaged. Consult instructions for use. Contains phthalates.

Monitoring Procedures

Independent monitoring of the trial for clinical protocol and compliance will be conducted periodically (i.e., at a minimum of three monthly intervals) by qualified staff from the the GCP unit in the Capital Region. The sponsor-investigator will permit direct access to the study monitors and appropriate regulatory authorities to the study data and to the corresponding source data and documents to verify the accuracy of these data.

Other treatments

Other treatments that are considered necessary for the subject will be given at the discretion of the investigator. Administration of all other treatments will be recorded on the accompanying Case Report Form.

Procedures in case of emergencies

The investigator will ensure that the necessary procedures and expertise to handle emergency situations that may arise during the study will be available.

Adverse Events

Adverse events (AEs) are defined as any event, sign or symptom occuring during the study whether the AE is considered to be related to the intervention or not. All adverse events must be recorded in the patient's Case Report Forms.

If an AE occurs more than 1 week after the removal of the PleurX catheter or more than 1 week after completion of Visit 8, and there is no apparent causal connection or relation to the Pleurx catheter, this shall not be deemed to be an AE.

The median survival for patients with cirrhosis and diuretic resistant ascites is six months and mortality is therefore expected amongst study participants. Consequently fatal events due to chronic liver failure will not be categorised as a SADE.

Furthermore the listed parameters below occur regularly amongst patients with decompensated cirrhosis and ascites. They are thoroughly evaluated and afterwards categorized, by specialized hepatologists, as not being associated with carrying a permanent tunnelated peritoneal catheter. As a result this list of events will not be categorised at SAE but will be registered as AE:

- Hospitalisation du to large volume paracenthesis
- Alterations in type and dose of medicine
- Planned outpatient control visits due to other chronical diseases
- Hospitalisations due to chronic liver failure
- Hospitalisations due to alcohol intoxication
- Diagnosis of Hepatocellular carcinoma or other malignant diseases

Some side effects of ascites are obtained by letting the study participants answer the Quality of Life Questionnaire at every visit. Therefore these side effects will not also be listed as Adverse Events:

- Stomach pain or soreness
- Nausea
- Anorexia
- Reduced appetite
- Alterations in bowel habits
- Fatigue
- Dyspnoea
- Negative alterations in physical capability
- Leg pain

The type, potential relationship, severity, starts and end date for all AE must be recorded. The severity of the adverse event and the relationship with the study medical device must be assessed in accordance with the guidelines described below.

*The investigator must assess the relationship between an adverse event and the study medical device using the following guidelines:*

Adverse Device Effect (ADE): an adverse event related to the use of the medical device

Serious Adverse Event (SAE / pre-event): an event that results in i) death; ii) life-threatening injury or illness; iii) lasting harm to the body or bodily functions; iv) hospitalization or prolongation of existing hospitalization or required medical or surgical treatment are necessary to prevent i to iv.

Serious Adverse Device Effect (SADE): a serious incident which is related to the use of the medical device.

An almost-event is an equipment incident which does not have a serious output because of intervention before the event has evolved.

Serious incidents and near-incidents caused by improper treatment or error due to technical errors or defects in the equipment, instructions, labelling, use or maintenance of the equipment.

A summary of product characteristics of the PleurX Peritoneal Catheter listed in the Investigational Brochure will be used as a reference document when it is assessed whether a serious related adverse event is expected or unexpected.

Grading of adverse events:

The investigator should attempt to identify all clinical and objective reactions from subjects and determine their relationship with the use of the allocated intervention (PleurX tunnelated catheter or repeated large volume paracentesis). Reactions, if there are any, should be graded according to the following scale:

1 = slight

2 = moderate

3 = severe

4 = life-threatening

Reporting of side effects of AEs and SAEs/SADEs

The investigator is responsible for ensuring that all adverse events are recorded in the patient's Case Report Form.

The sponsor is responsible for ongoing monitoring of the study's risk/benefit relation. The following must always be always immediately reported to the Danish Medicines Agency: any potential SAEs/SADEs and any situation that may affect the safety of the study subjects or the performance of the study. Similarly, reports should be made to all investigators and the Research Ethics Committees involved.

The sponsor shall at all time be kept informed by the investigator of any adverse events. At the end of the trial, the final report shall contain a description of all side effects.

Serious Adverse Device effect (SADE): Should be reported immediately by the investigator to the sponsor (sponsor-investigator). SADEs must be reported annually by the Investigator to the Research Ethics Committee throughout the whole trial period, together with a report on the safety of the trial subjects.

Fatal or life-threatening SADEs must be reported within 7 days of the sponsor becoming aware of them, and within 8 days of the report the sponsor must inform the Danish Medicines Agency of all relevant information on the follow-up.

All other SADEs should be reported to the same authorities within 15 days of the sponsor (sponsor-investigator) being informed of these.

All reports must be accompanied by comments on any consequences there are for the trial.

The sponsor (sponsor-investigator) will also inform the manufacturers of the medical device.

STATISTICS AND DATA MANAGEMENT

Sample Size Considerations

The primary outcome measure is paracentesis free survival. With an estimated probability of paracentesis free survival set to 0.3 at the end of the trial, an allocation ratio of 1:1, alpha 5% and power 80%, the required sample size is estimated to be a total of 28 patients (14 patients in each group). After taking into account the risk of participant dropout the sample size is set to be 32 patients (16 patients in each group).

Statistical analyses

We plan to include all patients randomised (intention to treat) regardless of compliance or follow up. Patient characteristics will be summarised using medians with interquartile range or proportions. Groups will be compared using t-tests or chi-square tests as appropriate. The level of significance will be set to 5%. The primary outcome measure will be evaluated using Kaplan Meier Plots and log-rank tests. Univariate and multivariate logistic regression analysis will be conducted to identify potential predictors of paracentesis free survival.

Handling of data

Data will be retrieved from medical records, information provided by the patients at trial visits as well as laboratory (biochemistry) assessments and investigational results. Data will be collected to Case report forms and entered into an electronic database by the principal investigator. Case report forms from the supporting sites will be sent by secure mail to the principal investigator. The database is anonymous. A trial participant identification list will be stored safely by the principal investigator.

Missing data

During the trial, we plan to search for information about missing data in the medical records. Investigators will pursue the options of repeating biochemistry and blood analyses when this is possible. Invasive procedures will not be repeated, should data be missing after the participant has completed the trial investigations.

Quality Control

The study personnel will be trained and tested in all study related procedures to ensure adequate quality control. In particular, investigators will be tested 0to ensure the optimal use of the interventions assessed (PleurX and large volume paracentesis). All investigators are specialised hepatologists with extensive experience in the management and observation of patients with refractory ascites including experience in the use of the PleurX and large volume paracentesis through their clinical work.

Data registration and rules for the control of investigation procedures

The study will be conducted in accordance with the applicable rules regarding quality control and quality management in clinical trials involving people and will follow EN ISO 14155- ”Clinical investigation of medical devices for human subjects – Good clinical practice.

The investigator and co-investigators at the clinical sites are responsible for managing and archiving data in accordance with current regulations. The data belongs to the investigator and co-investigators at the Gastro Unit at Hvidovre Copenhagen University Hospital.

Case Report Forms

A Case Report Form (CRF) will be completed for each patient included in the study. This will be signed by the investigator to confirm the accuracy of the data. Corrections to the data will only be made by crossing out the incorrect data (the incorrect information will remain visible and legible) with the correct data written next to the deleted data. Correction fluid will not be used. Corrections will be dated and signed by the investigator or their deputy.

Monitoring

Copenhagen University Hospital GCP unit

Bispebjerg Hospital, Building 51, 3rd floor

Bispebjerg Bakke 23

2400 Copenhagen NV

Tel. 3531 3890

Training

The investigator will ensure that the personnel involved are appropriately trained and qualified, and have the necessary information to carry out the study.

PATIENT SAFETY AND ETHICS

Informed Consent Process

The informed consent process will include both written and oral information using the prespecified forms. We will review and discuss risks and possible benefits of study participation with participants and their families. An approved consent form describing in detail the study procedures and risks will be given to the participant. The participant is required to read and review the document or have the document read to him or her. The investigator or designee will explain the research study to the participant and answer any questions that may arise. The participant will sign the informed consent document prior to any study-related assessments or procedures. Participants will be given the opportunity to discuss the study with their family or think about it prior to agreeing to participate. They may withdraw consent at any time throughout the course of the study. A copy of the signed informed consent document will be given to participants for their own records.

Participant Confidentiality

The participants will sign a consent form that allows investigators access to hospital records. Participant confidentiality is extended to cover any study information relating to participants. The study protocol, documentation, data, and all other information generated will be held in strict confidence. No information concerning the study or the data will be released to any unauthorized third party. Study investigators and scientific medical advisers will have access to study data. When conveying study materiel to external data assessors the materiel will be anonymized.

Study Records Retention

Study records will be maintained for at least five years from the date that the study is completed.

Respect for the patients physical and mental integrity and privacy

This study will be conducted according to the Danish national legislation on health. The study will be reported to the Regional Science Ethics Committee of the capital region of Denmark. The permission from The Regional Data Protection Agency will be sought and is awaited before the initiation of the study. Authorities will have full access to data, documents and registration procedures during monitoring, audits and inspections. Both oral and written informed consent will be obtained before entering the study according to the Declaration of Helsinki V and the Regional Science Ethics Committee of the capital region of Denmark. The study will comply with the law on personal data.

Ethical considerations

The PleurX catheter has proven efficient in the handling of malignant ascites by relieving symptoms and improving quality of life in this patient group. Our main aim is to give patients with ascites due to cirrhosis the same options in handling an invalidating complication to their disease, and achieve higher impact and involvement in the disease course. We also aim to diminish number of days spent in hospital and thereby adding to quality of life and well-being of the patient. The discomfort applied on the patient at the PleurX catheter placement exceeds that of a single standard paracentesis. However, all participants have resistant, recurrent ascites and are in need of repeated paracenteses weekly, bimonthly or monthly. Therefore, we consider the PleurX catheter to be a relatively proper and safe option for participants.

FINANCES AND INSURANCE

Insurance

Trial participants will be insured through the national patient insurance. Trial participants are informed of this relation in written information of trial participant’s rights, appendix 2. Sponsor and investigators are covered by their employment hospitals statutory insurance.

Finance and funding

PleurX catheters and drainage bottles will be purchased from Vingmed Danmark A/S. Drainage bottles for participants will be provided by Carefusion BD as a grant for device (up to 800 bottles). Carefusion BD does not provide financial support in addition to devices. Gastro Unit, medical division, Amager and Hvidovre Hospital will incur inpatient expenses during the trial.

Vingmed Danmark A/S is not further involved in the trial and has no influence whatsoever on the design and methodology, nor on compilation and publication of results.

Sponsor and initiator Lise Lotte Gluud has invented the overall template of this research project and has taken initiative to execute it. None of the involved investigators have economic or commercial conflicts regarding the investigations.

Investigators will be financed through clinical employments at their respective hospitals.

Expenses regarding trial investigations, admissions to hospital for the purpose of participating in the trial and extra outpatient visits will be undertaken by the involved hospitals.

Additional costs and expenses, including biochemical analyses and investigations will be covered through funds.

The trial has received grants from: University Hospital Hvidovre Research Foundation (2015; 84.000 Dkr.), Copenhagen University Internationalization Fund (2016; 120.000 Dkr.) and Hvidovre Hospitals Lægefond til bekæmpelse af Leversygdomme (2016; 30.000 Dkr).

PUBLICATION

The trial will be registered in clinical trial database www.clinicaltrials.gov before inclusion of the first patient. The results of the trial will be published in medical journals and conferences regardless of the results (positive, negative or inconclusive). We expect that the trial will lead to at least one publication. If we are unable to publish the result in a scientific journal, the results of the trial will be made public on www.clinicaltrials.gov.

LIST OF APPENDICES

Appendix A: Investigational programme (English)

Appendix B: Protocol resume in Danish

Appendix C: Participant’s information (deltagerinformation in Danish)

Appendix D: Informed consent form (Danish)

Appendix E: Proxy statement (Danish)

Appendix F: Pictures of the equipment (PleurX Brochure)

Appendix G: Quality of life questionnaire for patients with cirrhosis and ascites.

# REFERENCES

1. Planas R, Montoliu S, Balleste B, et al. Natural history of patients hospitalized for management of cirrhotic ascites. Clin Gastroenterol Hepatol 2006;**4**(11):1385-94.

2. EASL clinical practice guidelines on the management of ascites, spontaneous bacterial peritonitis, and hepatorenal syndrome in cirrhosis. J Hepatol 2010;**53**(3):397-417.

3. Gines P, Quintero E, Arroyo V, et al. Compensated cirrhosis: natural history and prognostic factors. Hepatology 1987;**7**(1):122-8.

4. Marchesini G, Bianchi G, Amodio P, et al. Factors associated with poor health-related quality of life of patients with cirrhosis. Gastroenterology 2001;**120**(1):170-8.

5. Llach J, Gines P, Arroyo V, et al. Prognostic value of arterial pressure, endogenous vasoactive systems, and renal function in cirrhotic patients admitted to the hospital for the treatment of ascites. Gastroenterology 1988;**94**(2):482-7.

6. Arroyo V, Gines P, Gerbes AL, et al. Definition and diagnostic criteria of refractory ascites and hepatorenal syndrome in cirrhosis. International Ascites Club. Hepatology 1996;**23**(1):164-76.

7. Runyon BA. Management of adult patients with ascites due to cirrhosis. Hepatology 2004;**39**(3):841-56.

8. Salerno F, Borroni G, Moser P, et al. Survival and prognostic factors of cirrhotic patients with ascites: a study of 134 outpatients. Am J Gastroenterol 1993;**88**(4):514-9.

9. Guardiola J, Xiol X, Escriba JM, et al. Prognosis assessment of cirrhotic patients with refractory ascites treated with a peritoneovenous shunt. Am J Gastroenterol 1995;**90**(12):2097-102.

10. Bajaj JS, O'Leary JG, Reddy KR, et al. Survival in infection-related acute-on-chronic liver failure is defined by extrahepatic organ failures. Hepatology 2014;**60**(1):250-6.

11. Garcia-Tsao G. Current management of the complications of cirrhosis and portal hypertension: variceal hemorrhage, ascites, and spontaneous bacterial peritonitis. Gastroenterology 2001;**120**(3):726-48.

12. Rimola A, Garcia-Tsao G, Navasa M, et al. Diagnosis, treatment and prophylaxis of spontaneous bacterial peritonitis: a consensus document. International Ascites Club. J Hepatol 2000;**32**(1):142-53.

13. Tandon P, Garcia-Tsao G. Bacterial infections, sepsis, and multiorgan failure in cirrhosis. Semin Liver Dis 2008;**28**(1):26-42.

14. Belcher JM, Garcia-Tsao G, Sanyal AJ, et al. Association of AKI with mortality and complications in hospitalized patients with cirrhosis. Hepatology 2013;**57**(2):753-62.

15. John S, Thuluvath PJ. Hyponatremia in cirrhosis: pathophysiology and management. World J Gastroenterol 2015;**21**(11):3197-205.

16. Maruyama H, Kondo T, Kiyono S, et al. Relationship and interaction between serum sodium concentration and portal hemodynamics in patients with cirrhosis. J Gastroenterol Hepatol 2015.

17. Maruyama H, Kondo T, Sekimoto T, et al. Hyponatremia: a significant factor in a poor prognosis for cirrhosis with Child A/B after variceal eradication. J Hepatobiliary Pancreat Sci 2015.

18. Adebayo D, Morabito V, Davenport A, et al. Renal dysfunction in cirrhosis is not just a vasomotor nephropathy. Kidney Int 2015;**87**(3):509-15.

19. Bozanich NK, Kwo PY. Renal insufficiency in the patient with chronic liver disease. Clin Liver Dis 2015;**19**(1):45-56.

20. Low G, Alexander GJ, Lomas DJ. Hepatorenal syndrome: aetiology, diagnosis, and treatment. Gastroenterol Res Pract 2015;**2015**:207012.

21. Moller S, Dumcke CW, Krag A. The heart and the liver. Expert Rev Gastroenterol Hepatol 2009;**3**(1):51-64.

22. Moller S, Henriksen JH. Cardiovascular complications of cirrhosis. Gut 2008;**57**(2):268-78.

23. Moller S, Henriksen JH. Cardiovascular complications of cirrhosis. Postgrad Med J 2009;**85**(999):44-54.

24. Fasolato S, Angeli P, Dallagnese L, et al. Renal failure and bacterial infections in patients with cirrhosis: epidemiology and clinical features. Hepatology 2007;**45**(1):223-9.

25. Gines P, Schrier RW. Renal failure in cirrhosis. N Engl J Med 2009;**361**(13):1279-90.

26. Maharshi S, Sharma BC, Srivastava S. Malnutrition in Cirrhosis Increases Morbidity and Mortality. J Gastroenterol Hepatol 2015.

27. Merli M, Riggio O, Dally L. Does malnutrition affect survival in cirrhosis? PINC (Policentrica Italiana Nutrizione Cirrosi). Hepatology 1996;**23**(5):1041-6.

28. Campillo B, Bories PN, Pornin B, et al. Influence of liver failure, ascites, and energy expenditure on the response to oral nutrition in alcoholic liver cirrhosis. Nutrition 1997;**13**(7-8):613-21.

29. Stein TP. Nutrition and protein turnover: a review. JPEN J Parenter Enteral Nutr 1982;**6**(5):444-54.

30. Grimble RF. Nutritional modulation of immune function. Proc Nutr Soc 2001;**60**(3):389-97.

31. Suchner U, Kuhn KS, Furst P. The scientific basis of immunonutrition. Proc Nutr Soc 2000;**59**(4):553-63.

32. Kalman DR, Saltzman JR. Nutrition status predicts survival in cirrhosis. Nutr Rev 1996;**54**(7):217-9.

33. Alberino F, Gatta A, Amodio P, et al. Nutrition and survival in patients with liver cirrhosis. Nutrition 2001;**17**(6):445-50.

34. Thiele M, Askgaard G, Timm HB, et al. Predictors of health-related quality of life in outpatients with cirrhosis: results from a prospective cohort. Hepat Res Treat 2013;**2013**:479639.

35. Thomsen KL, Sandahl TD, Holland-Fischer P, et al. Changes in adipokines after transjugular intrahepatic porto-systemic shunt indicate an anabolic shift in metabolism. Clin Nutr 2012;**31**(6):940-5.

36. Bernardi M, Laffi G, Salvagnini M, et al. Efficacy and safety of the stepped care medical treatment of ascites in liver cirrhosis: a randomized controlled clinical trial comparing two diets with different sodium content. Liver 1993;**13**(3):156-62.

37. Angeli P, Gatta A, Caregaro L, et al. Tubular site of renal sodium retention in ascitic liver cirrhosis evaluated by lithium clearance. Eur J Clin Invest 1990;**20**(1):111-7.

38. Angeli P, Volpin R, Piovan D, et al. Acute effects of the oral administration of midodrine, an alpha-adrenergic agonist, on renal hemodynamics and renal function in cirrhotic patients with ascites. Hepatology 1998;**28**(4):937-43.

39. Angeli P, Fasolato S, Mazza E, et al. Combined versus sequential diuretic treatment of ascites in non-azotaemic patients with cirrhosis: results of an open randomised clinical trial. Gut 2010;**59**(1):98-104.

40. Santos J, Planas R, Pardo A, et al. Spironolactone alone or in combination with furosemide in the treatment of moderate ascites in nonazotemic cirrhosis. A randomized comparative study of efficacy and safety. J Hepatol 2003;**39**(2):187-92.

41. Singh V, Dhungana SP, Singh B, et al. Midodrine in patients with cirrhosis and refractory or recurrent ascites: a randomized pilot study. J Hepatol 2012;**56**(2):348-54.

42. Sanyal AJ, Genning C, Reddy KR, et al. The North American Study for the Treatment of Refractory Ascites. Gastroenterology 2003;**124**(3):634-41.

43. Gines P, Uriz J, Calahorra B, et al. Transjugular intrahepatic portosystemic shunting versus paracentesis plus albumin for refractory ascites in cirrhosis. Gastroenterology 2002;**123**(6):1839-47.

44. Montomoli J, Holland-Fischer P, Bianchi G, et al. Body composition changes after transjugular intrahepatic portosystemic shunt in patients with cirrhosis. World J Gastroenterol 2010;**16**(3):348-53.

45. Saab S, Nieto JM, Lewis SK, et al. TIPS versus paracentesis for cirrhotic patients with refractory ascites. Cochrane Database Syst Rev 2006(4):CD004889.

46. Salerno F, Merli M, Riggio O, et al. Randomized controlled study of TIPS versus paracentesis plus albumin in cirrhosis with severe ascites. Hepatology 2004;**40**(3):629-35.

47. Sharzehi K, Jain V, Naveed A, et al. Hemorrhagic complications of paracentesis: a systematic review of the literature. Gastroenterol Res Pract 2014;**2014**:985141.

48. Luca A, Feu F, Garcia-Pagan JC, et al. Favorable effects of total paracentesis on splanchnic hemodynamics in cirrhotic patients with tense ascites. Hepatology 1994;**20**(1 Pt 1):30-3.

49. Pozzi M, Osculati G, Boari G, et al. Time course of circulatory and humoral effects of rapid total paracentesis in cirrhotic patients with tense, refractory ascites. Gastroenterology 1994;**106**(3):709-19.

50. Bellot P, Welker MW, Soriano G, et al. Automated low flow pump system for the treatment of refractory ascites: a multi-center safety and efficacy study. J Hepatol 2013;**58**(5):922-7.

51. White J, Carolan-Rees G. PleurX peritoneal catheter drainage system for vacuum-assisted drainage of treatment-resistant, recurrent malignant ascites: a NICE Medical Technology Guidance. Appl Health Econ Health Policy 2012;**10**(5):299-308.

52. Rosenberg S, Courtney A, Nemcek AA, Jr., et al. Comparison of percutaneous management techniques for recurrent malignant ascites. J Vasc Interv Radiol 2004;**15**(10):1129-31.

53. Kathpalia P, Bhatia A, Robertazzi S, et al. Indwelling Peritoneal Catheters in Patients with Cirrhosis and Refractory Ascites. Intern Med J 2015.
