# Supplementary figures and images for: Tunneled Peritoneal Catheter for Refractory Ascites in Cirrhosis: A Randomized Case-Series
Source: Medicina (Kaunas). 2020 Oct 27;56(11):565. doi: 10.3390/medicina56110565 (PMC7692861; doi:10.3390/medicina56110565)

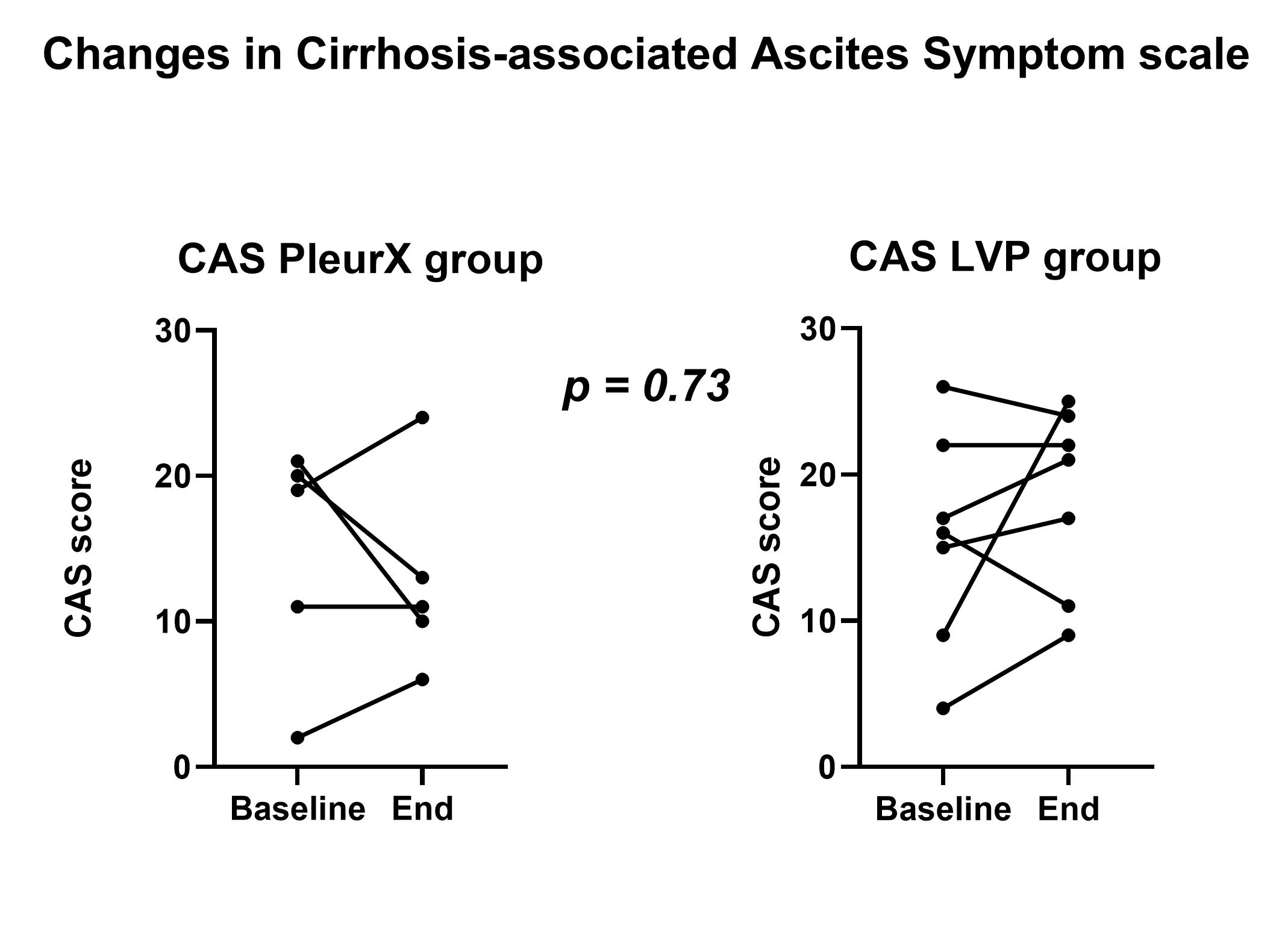

Supplement: Supplementary file 1 [file medicina-56-00565-s001.zip › Suppl Fig 1 Cirrhosis Ascites Symptom Score.jpg]
